# Meeting report

**DOI:** 10.1155/S1463924600000031

**Published:** 2000


with other researchers, industrial development engineers and instrumentation manufacturers to ensure continuous product development, not only in lamps but also in ancillaries and systems, e.g. the new Fiberlight light source. This total capability ensures that manufacturers and users of analytical instrumentation achieve optimum performance from highly sophisticated equipment.

Heraeus Noblelight Ltd, part of the Heraeus Group, specializes in the production and application of high-quality energy sources covering the entire electromagnetic spectrum, from ultraviolet to infra-red. It offers a range of UV lamps to match most of the UV/VIS Spectrometers and HPLC UV Detectors in use today.

*For more information contact Martin Woods, Heraeus Noblelight Ltd, Cambridge Science Park, Milton Road, Cambridge CB4 4GQ, UK. Tel: +44 (0) 1223 423324, Fax: +44 (0) 1223 423999*

### Meeting report

***P S Analytical sets the standard at Pittcon 99***

At the Pittsburgh Conference in Orlando, Florida, P S Analytical introduced a number of exciting new products within its Millennium and Sir Galahad product ranges.

The Millennium Merlin/Galahad instrument has been introduced to fulfil the expectations of the EPA 631 methodology. The PSA 10.025 encorporates a gold amalgamation step into the already proven Millennium Merlin. The instrument can be used with either the standard PSA carousel autosampler or the new xyz model. The introduction of the amalgamation step increases the detection capabilities of the Merlin system with a sample throughput of ~10 samples per hour.

The standard Millennium Merlin has a detection range of <0.2 ppt quantity at a sample throughput of 30 samples per hour. One further unique features of this range is the ability to analyse samples in the 0–10 ppm range without any system modification.

Mercury, arsenic and selenium speciation are fast becoming significant, not only in the academic research area but also for future regulatory issues. The Millennium range of products can be readily integrated into chromatographic or purge-and-trap schemes to develop speciation profiles. To enable the products to do this, PSA has developed a simple retrofit 0–1 V option for the Millennium range, and is also able to provide the users with standard kits to enable speciation studies to be readily achieved.

In the petrochemical industry, there is also considerable interest in the determining presence of mercury either as mercury(0) or organic derivations. P S Analytical also introduced its PSA 10.515 preconcentration device to assist in the analysis of mercury levels in petrochemical condensates. This new instrument can handle 0–1 µl samples of condensates, and prepares samples prior to analysis by the PSA Sir Galahad instrument. Speciation profiles can be readily provided using the PSA 10.727 gas chromatography–atomic fluoresence system.

*Details of all of these new products are available from P S Analytical, Arthur House, Crayfields Industrial Estate, Main Road, Orpington, Kent BR5 3HP, UK. Tel: +44 (0)1689 891211; Fax: +44 (0)1689 896009; e-mail: sales@psanalytical.demon.co.uk; URL—http://www.psanalytical.com*

